# Emotion regulation in euthymic bipolar patients in Tunisia

**DOI:** 10.1192/j.eurpsy.2021.1665

**Published:** 2021-08-13

**Authors:** N. Messedi, E. Mhiri, F. Charfeddine, W. Bouattour, L. Aribi, J. Aloulou

**Affiliations:** 1 Psychiatry (b), Hedi Chaker University hospital, sfax, Tunisia; 2 Psychiatry (b), Hedi Chaker University hospital, Sfax, Tunisia

**Keywords:** remitted, emotion, regulation, bipolar

## Abstract

**Introduction:**

Bipolar disorder is presumed to involve difficulties in emotion regulation. However, little is known about the specific emotion regulation profile associated with this disorder.

**Objectives:**

To study emotion regulation in bipolar patients in remission phase and to determine the factors correlated with it.

**Methods:**

A cross-sectional, descriptive and analytical study of 30 patients followed for bipolar disorder in remission, at the psychiatric outpatient clinic at the Hédi Chaker University Hospital in Sfax. We used a socio-demographic and clinical data sheet and the Cognitive Emotion Regulation Questionnaire (CERQ) which assesses cognitive strategies (maladaptive and adaptive) for regulating emotion.

**Results:**

The mean age of the patients was 43.77 years, the sex ratio was 0.5. Bipolar I disorder was diagnosed in 93% of patients. A good adherence to treatment was found in 86.7% of cases and a good social integration in 40%. The mean total score of the adaptive CERQ was 66.73 and the most used adaptive strategy was acceptation (mean score =13.87), while the mean total score of the maladaptive CERQ was 36.7 and the most used maladaptive strategy was self blame (mean score =9.47). Adaptive cognitive emotion regulation was predominant in 93.3% of patients. It was significantly correlated with good adherence to treatment (p = 0.047) and good social integration (p = 0.026).

**Conclusions:**

Our patients with euthymic bipolar disorder showed a satisfying level of adaptive emotion regulation strategies. A cognitive remediation seems important to embetter this capacity and improve the income of the disease.

**Disclosure:**

No significant relationships.

